# Anterior peri-sacroiliac joint osteotomy in the treatment of malunion and nonunion of complex pelvic ring fracture: techniques and preliminary results

**DOI:** 10.1007/s00264-024-06173-w

**Published:** 2024-04-17

**Authors:** Shicai Fan, Yilan Liao, Zhipeng Xiao, Yuhui Chen, Tao Li, Qiguang Mai, Sheqiang Chen, Haibo Xiang, Zhiyong Hou, Yingze Zhang

**Affiliations:** 1 Trauma Emergency Center, the Third Hospital of Hebei Medical University, Shijiazhuang, 050051 China; 2 Department of Traumatic Surgery, Center for Orthopedic Surgery, the Third Affiliated Hospital of Southern Medical University, Guangzhou, 510630 China

**Keywords:** Pelvic fractures, Malunion, Nonunion, Anterior peri-sacroiliac joint osteotomy

## Abstract

**Purpose:**

To introduce anterior peri-sacroiliac joint osteotomy (APSJO) through the lateral-rectus approach (LRA) for treating pelvic fracture malunion and nonunion, and to evaluate the safety, feasibility, and potential effectiveness.

**Methods:**

Data of 15 patients with pelvic fracture malunion and nonunion who underwent treatment by APSJO were selected and analyzed. The reduction quality was assessed using the Mears and Velyvis criteria, while the pre-operative and post-operative function was revealed by the Majeed scoring system. The British Medical Research Council (BMRC) grading system was recruited for the evaluation of lumbosacral plexus function.

**Results:**

The average operative duration was 264.00 ± 86.75 min, while the intra-operative blood loss was 2000 (600, 3000) mL. Anatomical reduction was complete in three cases, satisfactory in ten cases, and unsatisfactory in two cases. Among the seven patients with lumbosacral plexus injury, the pre-operative Majeed grades were good in two cases, fair in two cases, and poor in three cases, while the post-operative Majeed grades were excellent in three cases, good in three cases, and fair in one case. Muscle strength recovered to M5 in two cases, M4 in three cases, and showed no recovery in two cases. The pre-operative Majeed grades were good in five cases, fair in two cases, and poor in one case of the series without lumbosacral plexus injury, while the post-operative Majeed grades were excellent in seven cases and good in one case.

**Conclusion:**

APSJO through LRA may be a feasible strategy for treating pelvic fracture malunion and nonunion with promising application.

## Introduction

Pelvic fracture is a severe trauma which is frequently associated with chest, abdomen, haemodynamic instability, and fracture malunion and nonunion is presented once the life-threaten injury is stable [1–3]. A high incidence of disability and deformity of the lower limbs results from the malunion and nonunion of a pelvic fracture [4, 5]. Due to the fracture displacement, callus formation, and soft tissue contracture, the anatomical structure cannot be distinguished in cases of pelvic fracture malunion and nonunion [6–8]. The surgical repair of delayed pelvic fracture is challenging for even senior surgeons, especially when the posterior ring is involved.

The most widely reported surgical treatment for pelvic fracture malunion and nonunion is the three-stage or two-stage surgery with a combined anterior and posterior approach [9, 10]. The iliac wing or the sacral osteotomy is the alternative way to correct posterior ring deformity [5, 7]. However, the complex procedures involved in the operation and the inadequate surgical exposure limit the effectiveness of surgical outcomes, and a high risk of complications has also been reported in three-stage or two-stage operations [7, 10]. A sufficient exposure is still needed to achieve a satisfactory correction of pelvic deformity.

In the previous study, the lateral-rectus approach (LRA) was used to conduct the repair of posterior pelvic ring injury and lumbosacral plexus injury [11]. The sacroiliac joint can be accessed easily by this approach. Hence, in the current investigation, the anterior peri-sacroiliac joint osteotomy (APSJO) through the LRA was used for the treatment of pelvic fracture malunion and nonunion. The feasibility, safety, and potential effectiveness of APSJO through the LRA were revealed simultaneously.

## Materials and methods

Our study was approved by the Ethics Committee of the Third Affiliated Hospital of Southern Medical University (2021–008). Informed consent was obtained from all included participants.

### Inclusion and exclusion criteria

From January 2017, surgical treatment of complex pelvic ring fractures by APSJO was initialed in our institution. Forty-three cases were included according to the following inclusion criteria: (1) pelvic malunion and nonunion; (2) vertical and/or rotational deformities originating from posterior pelvic ring injuries; (3) patients with disability due to the pelvic deformity; (4) age under 60 years. Forty-three cases were included.

Twenty-eight cases were excluded according to the exclusion criteria: (1) patients underwent iliac wing or sacral osteotomy, as well as conservative treatment; (2) incomplete follow-up time of less than 12 months; (3) patients with serious systemic diseases or unstable vital signs that cannot tolerate anaesthesia and operation; (4) severe mental illness precluding cooperation with surgical management.

### Pre-operative management

All patients underwent regular imaging examinations, including pelvic anteroposterior (AP), inlet and outlet X-ray, and CT scan (Siemens, Germany), to evaluate the deformity of the pelvic ring and determine the individualized osteotomy techniques. Computer modeling and 3D printing were utilized as needed. To analyze whether a patient’s pain was neurogenic or due to fracture instability, detailed physical examination, electromyography, and lumbosacral plexus magnetic resonance neurography (MRN) were performed when necessary to clarify the location and cause of sacroiliac pain, as well as its relationship with pelvic stability and deformity. Direct cross-matched blood and cell saver was prepared in all cases pre-operatively.

### Operative procedure

**Fig. 1:**
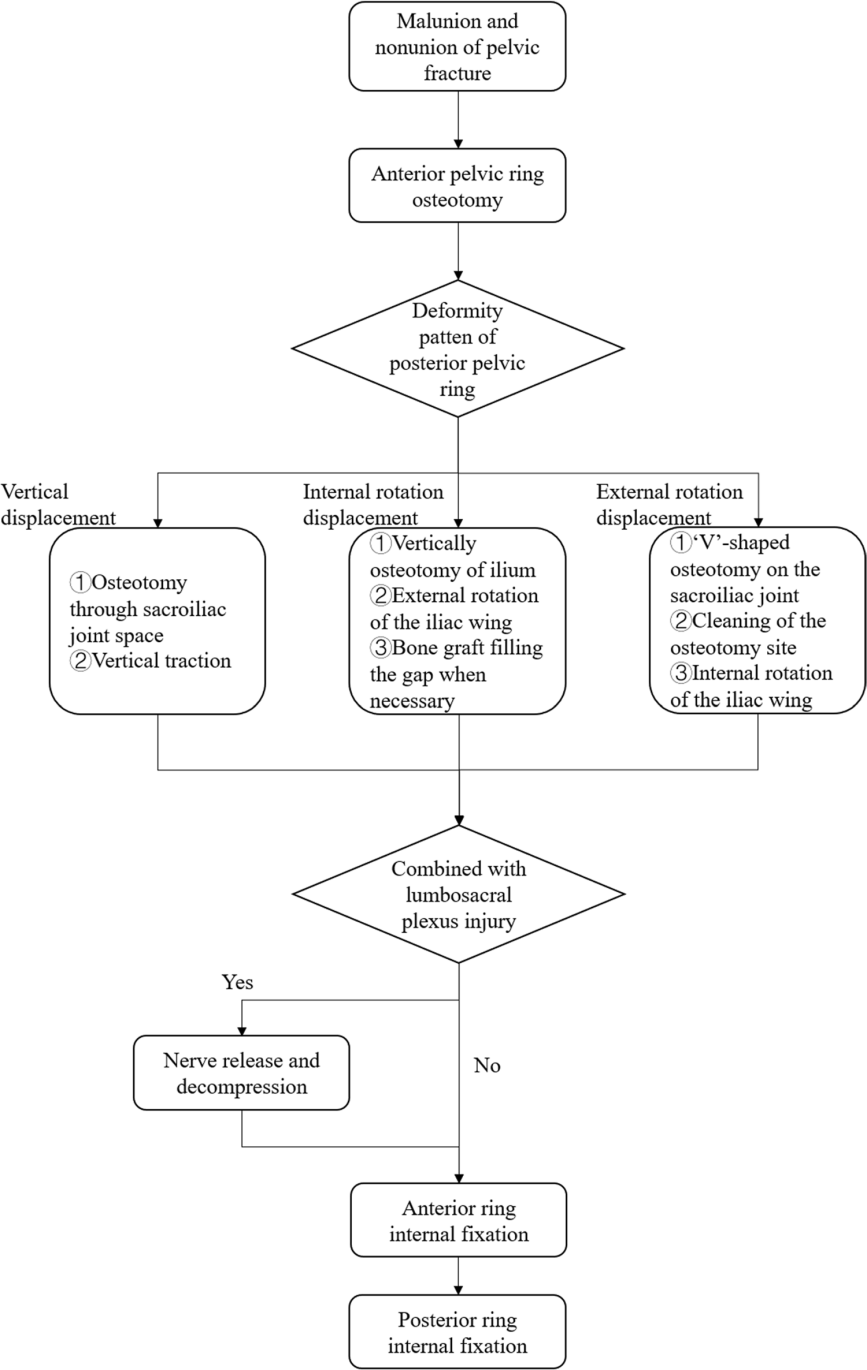
The surgical procedure

**Fig. 2:**
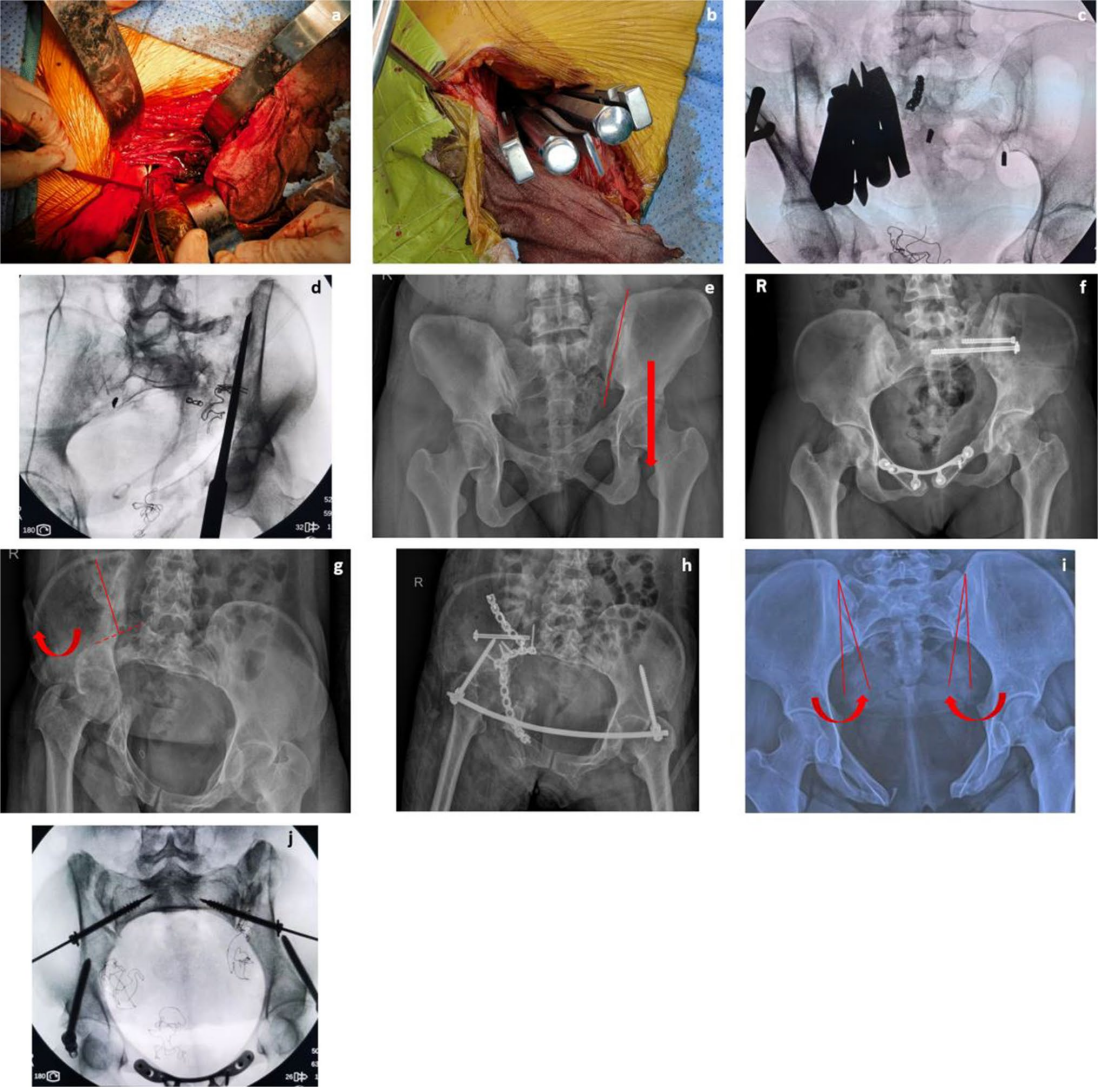
**a** The middle window of LRA exposed the structure around the sacroiliac joint and lumbosacral plexus, osteotomy, and lumbosacral plexus release were performed through this window. **b**, **c** The sacroiliac joint was separated after osteotomy by osteotome and hammer. **d** Bone cut using an osteotome along the osteotomy line. **e**, **f** Female, 31 years old, fall from height, vertical displacement corrected after the release of the sacroiliac joint and fixation with sacroiliac screws; the anterior pelvic ring was fixed using a pubic symphysis orthotic compression anatomic plate (PSOCAP, designed by our team for reduction of pubic symphysis) (Double Medical Technology Inc., Xiamen, China). **g**, **h** Female, 52 years old, injury caused by a traffic accident; internal rotation of the ilium was externally rotated using a Schanz screw after osteotomy and fixed with reconstruction plates. **i**, **j** Female, 33 years old, diastasis of the pubic symphysis caused by a traffic accident 12 years previously, with recent complaints of soreness of the symphysis and sacroiliac joint after giving birth. A “V”-shape osteotomy was performed on both sides, and sacroiliac screws and a PSOCAP were used for fixation

Tracheal intubation under general anaesthesia was performed, with the patient positioned in a supine position on a radiolucent operating table after routine disinfection. All surgical procedures were carried out by the same surgeon. Figure 1 shows the key surgical procedures. The typical case is shown in Fig. 2.

#### Exposure and osteotomy of the anterior pelvic ring

The anterior pelvic ring was exposed using the interior window of the LRA [15]. The obturator nerve and vessels were fully exposed and protected. Anterior pelvic ring osteotomy was performed following the pre-operative plan. The soft tissue around the osteotomy site was cleaned, and the anterior pelvic ring was fully released.

#### Exposure of the peri-sacroiliac joint and APSJO

The sacroiliac joint was exposed using the medial window of the LRA. The iliopsoas muscle and the external iliac artery and vein were separated, with the iliopsoas muscle pulled laterally, while the peritoneum, external iliac artery, and vein were pulled medially to expose the anterior aspect of the sacroiliac joint. The lumbosacral nerve and vascular structures around the medial sacral wing were fully protected during the exposure (Fig. 2a). Bipolar electrocoagulation was used to prevent bleeding from the anterior sacral venous plexus, and the sacroiliac joint was separated about 1 cm from the sacrum and ilium below the periosteum of the sacroiliac joint.

**Fig. 3:**
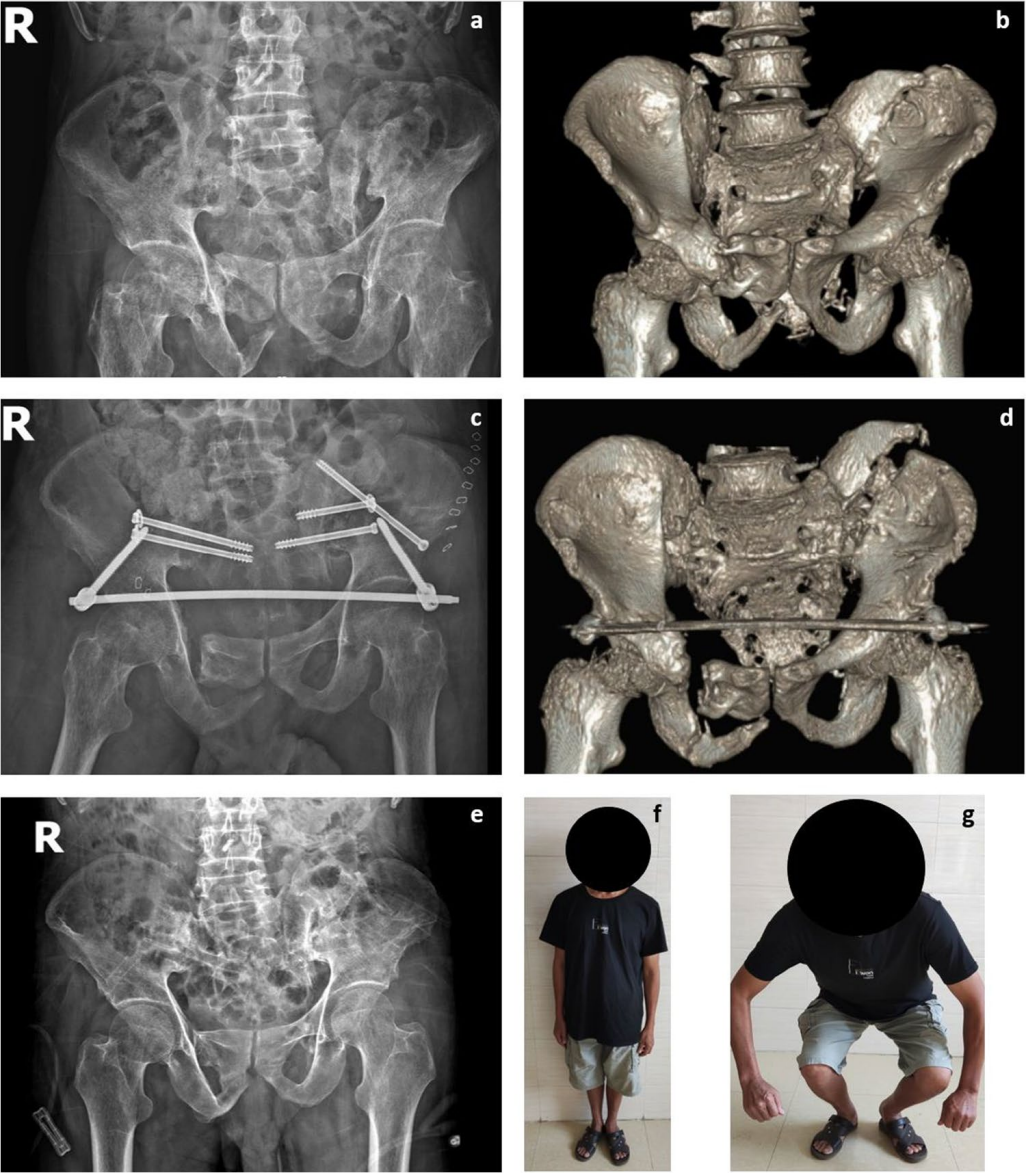
A case of complex deformity (male, 51 years old, injured by a car falling on him when repairing it under the chassis). The patient was sent to our institution 4 months after the injury, which was combined with pelvic infection and anal atresia after rectal colostomy, with a complaint of inability to stand and sit. Sacroiliac osteotomy was performed on both sides through the LRA. **a**, **b** Pre-operative AP X-ray view and 3D reconstruction of CT data. **c**, **d** Post-operative AP X-ray view and 3D reconstruction of CT data. **e** AP X-ray view at 2-year follow-up after removal of implants. **f**, **g** The patient’s quality of life was improved at the 2-year follow-up

Peri-sacroiliac osteotomy, reduction, and fixation were performed while ensuring the protection of the vessels and nerves on the medial side of the sacroiliac joint. Traditional tools such as osteotomes and hammers were used in the osteotomy procedure (Fig. 2b, c, d). The osteotomy plan was designed based on the displacement and deformity of the posterior ring: (1) In patients with vertical displacement, the sacroiliac joint space was completely separated using a wide osteotome, the surrounding soft tissue was released, and the vertical traction was conducted subsequently (Fig. 2e, f). (2) Internal rotation deformity was corrected by osteotomy on the ilium around the sacroiliac joint, with the direction of osteotomy being vertical to the iliac wing surface, followed by external rotation of the iliac wing to correct the deformity (Fig. 2g, h). (3) For external rotation deformity, a “V”-shaped osteotomy was performed on the sacroiliac joint, followed by cleaning of the osteotomy site and internal rotation of the iliac wing (Fig. 2i, j). In cases of complex pelvic ring deformities, a combination of the aforementioned methods was used (Fig. 3). The soft tissues around the osteotomy site of the sacroiliac joint were released, and an incision of the sacrospinous ligament through the ischial spine was performed if necessary. In cases where patients pre-operatively identified lumbosacral plexus injuries, release and decompression of the nerve were carried out through the middle window of the LRA [11]. When dealing with lumbosacral trunk injuries caused by soft tissue scar compression, it is essential to delicately remove the compressing soft tissue. In cases of bony compression, conducting osteotomy on the compressed bone while ensuring strict nerve protection can help alleviate nerve compression.

#### Reduction and fixation

After performing the osteotomy, lower limb traction or LC-II Schanz screws were used to achieve reduction. Starr frame could be used to overcome the tight pelvic floor muscles and assist reduction [12]. Bone grafting was carried out when necessary. We start internal fixation first anteriorly to help maintain the reduction and fixation of the posterior ring, on account of the high tension of the posterior ring deformity caused by fracture displacement, callus formation, and soft tissue contracture.

### Post-operative management

An indwelling drainage tube was used after the operation and was removed once the daily drainage volume was less than 50 mL. To prevent deep venous thrombosis in the lower limbs, Enoxaparin was routinely administered six hours after surgery. Non-weight-bearing activity was started four weeks after surgery. Full weight-bearing walking was allowed three months after fracture healing.

### Evaluation methods

#### Mears and Velyvis criteria

Post-operative pelvic fracture reduction quality was assessed according to the Mears and Velyvis criteria by post-operative AP, inlet and outlet X-rays, and CT scans [13]. The results were divided into three grades: anatomical (anatomical correction in all three radiological views), satisfactory (residual deformity of less than 1 cm of vertical and/or posterior displacement and/or less than 15° of rotational deformity in any plane), and unsatisfactory (residual deformity of greater than 1 cm of vertical or posterior displacement and/or more than 15° of rotational deformity).

#### Majeed scoring system

The Majeed scoring system was used to compare pre-operative and post-operative function at the post-operative follow-up, while the imaging reduction evaluation was not able to completely represent the functional status of the patient [14]. The scoring system consists of four parts: pain (30 points), work performance (20 points), sitting (10 points), standing (36 points), and sexual intercourse (4 points), to give a total of 100 points. The result was divided into four levels: excellent (≥ 85 points), good (70–84 points), fair (55–69 points), and poor (< 55 points).

#### British Medical Research Council grading system

The lumbosacral plexus function was evaluated using the British Medical Research Council (BMRC) grading system (used with the permission of the Medical Research Council) [15]. This scoring system is divided into six levels as follows: M0 (none) represents no evidence of contractility; M1 (trace) represents evidence of slight contractility but no joint motion, or return of perceptible contraction of the proximal muscles; M2 (poor) represents complete range of motion (ROM) with gravity eliminated, proximal muscle contraction against gravity, and perceptible distal muscle contraction; M3 (fair) represents complete ROM against gravity and return of function in proximal and distal muscles to such a degree that all important muscles are sufficiently powerful against gravity; M4 (good) represents complete ROM against gravity with some resistance, all muscles acting against strong resistance, and some independent movements are possible, but with some intrinsic weakness; M5 (normal) represents complete range of motion against gravity with full resistance and full recovery in all muscles. A post-operative grade of M5 or M4 was considered clinical recovery.

### Statistical analysis

The Shapiro–Wilk test was employed to test for normal distribution of continuous data. Variables that followed a normal distribution are presented as‾*x* ± *s*; others are presented as *M* (*P*_25_, *P*_75_). To compare pre-operative and post-operative function, the Wilcoxon two-sample rank sum test was employed for the comparison of Majeed and BMRC grades. A *p* value of < 0.05 was considered statistically significant. All statistical analyses were conducted using IBM SPSS 27.0 software (IBM Corp., Armonk, NY, USA).

## Results

From January 2017 to June 2022, data of 15 patients (seven males and eight females) with pelvic fracture malunion and nonunion who received surgical treatment involving APSJO were collected and analyzed (Table 1). The mean age of patients was 37.53 ± 13.27 years (range, 8–54 years). Eleven cases were the result of traffic accidents, two cases were caused by falls, and two cases were injuries caused by heavy objects, and the time from injury to operation was 4 (3, 9) months (range, 2–144 months). According to the AO/OTA classification, one case was type of B3, five cases were type C1.3, two cases were type C2, and seven cases were C3. Of these 15, seven cases were diagnosed with lumbosacral plexus injuries.

**Table 1:** Patient demographics and injury data

Case number	Gender	Age (years)	Mechanism of injury	Delay to surgery (months)	Tile classification	Lumbosacral plexus injury
1	Male	39	T	4	C1.3	Yes
2	Female	31	F	5	C1.3	
3	Male	47	T	17	C3	Yes
4	Male	54	H	5	C3	Yes
5	Male	33	F	11	C3	
6	Female	52	T	9	C2	Yes
7	Female	50	T	3	C3	Yes
8	Male	8	T	3	C1.3	Yes
9	Female	41	T	4	C3	
10	Female	33	T	144	B3	
11	Male	51	H	4	C3	
12	Male	21	T	3	C3	
13	Female	38	T	3	C1.3	
14	Female	21	T	3	C2	Yes
15	Female	44	T	2	C1.3	

*F* fall from height, *T* traffic accident, *H* injury caused by heavy objects

As shown in Table 2, all cases were followed up for at least 12 (12, 15) months (range, 12–24 months). The operative duration of patients was 264.00 ± 86.75 min (range, 155–475 min), while the intra-operative blood loss was 2000 (600, 3000) mL (range, 350–6300 mL). Thirteen patients required intra-operative blood transfusion. Fracture healing was observed at 4.53 ± 2.98 months (range, 2–8 months) post-operation.

**Table 2:** General surgical data and follow-up

Case number	Operative duration (min)	Intra-operative blood loss (mL)	Direct cross-matched blood transfusion (mL)	Autologous blood transfusion (mL)	Fracture healing time (months)	Follow-up (months)	Remarks
1	360	1000	800	0	2	12	Two operations reconstruction*
2	235	3000	800	1000	3	12	
3	475	6000	4400	0	6	15	Bedsore on sacrococcygeal region, post-operatively ICU management
4	240	3000	1200	800	3	24	Two operations reconstruction*
5	195	350	0	0	2	12	
6	355	6300	2800	400	8	15	Post-operatively ICU management
7	180	600	400	0	6	12	
8	160	700	1000	0	4	12	
9	310	3000	3800	0	6	12	
10	255	3000	1200	200	4	12	
11	240	400	800	160	4	14	
12	310	2500	2400	0	6	12	
13	275	400	0	0	4	12	
14	155	1200	800	0	4	12	
15	215	2000	2200	0	6	15	

^*^For cases which underwent a two-stage of operation, the operative statistics above were from the first stage

Anatomical reduction was complete in three cases, satisfactory in ten cases, and unsatisfactory in two cases in accord with the Mears and Velyvis criteria. Among the seven patients with lumbosacral plexus injury, the pre-operative Majeed grades were good in two cases, fair in two cases, and poor in three cases, while the post-operative Majeed grades were excellent in three cases, good in three cases, and fair in one case at the end of follow-up. The differences in Majeed grade between pre-operative and post-operative scores were statistically significant (*p* = 0.015). The pre-operative BMRC grades were M3 in one case, M2 in one case, M1 in two cases, and M0 in three cases (Table 3). Muscle strength of patients was significantly enhanced post-operatively (*p* = 0.017). The BMRC grades of patients were M5 in two cases and M4 in three cases, while no recovery in two cases (Table 3). Additionally, the pre-operative Majeed grades were good in five cases, fair in two cases, and poor in one case in the series without lumbosacral plexus injury, while the post-operative Majeed grades were excellent in seven cases and good in one case (Table 3). These data suggested a functional improvement of patients post-operatively (*p* = 0.009).

**Table 3:** Radiological and functional evaluation

Case number	Mears and Velyvis criteria	Majeed grade		BMRC grade	
		Pre-operative	Post-operative	Pre-operative	Post-operative
1	S	40 (poor)	82 (good)	M0	M1
2	A	68 (fair)	97 (excellent)		
3	U	43 (poor)	72 (good)	M0	M1
4	S	72 (good)	86 (excellent)	M3	M5
5	S	82 (good)	94 (excellent)		
6	S	52 (poor)	68 (fair)	M2	M4
7	S	68 (fair)	87 (excellent)	M0	M4
8	A	69 (fair)*	97 (excellent)*	M1	M5
9	S	66 (fair)	93 (excellent)		
10	A	70 (good)	96 (excellent)		
11	S	68 (fair)	89 (excellent)		
12	S	68 (fair)	87 (excellent)		
13	S	50 (poor)	84 (good)		
14	S	65 (fair)	77 (good)	M1	M4
15	S	83 (good)	94 (excellent)		

*A* anatomic reduction, *S* satisfactory reduction, *U* unsatisfactory reduction

^*^This 8-year-old boy was underage; consequently, the sexual intercourse item was allocated as a full score of 4

No surgical complications (such as iatrogenic neurovascular injury, wound infection, screw loosening) were presented in any patients during follow-up.

## Discussion

Conservative treatment of complex pelvic ring fracture malunion and nonunion usually results in disability and continuing pain. However, the treatment of pelvic fracture malunion and nonunion is challenging for most surgeons due to the limitation of operative exposure and the alterations of anatomic structures. As described in previous reports, by using the LRA, the sacroiliac joint, lumbosacral plexus, and blood vessels can be exposed and distinguished anteriorly [16]. Each APSJO was individually designed and performed on the patients with pelvic fracture malunion and nonunion. Moreover, this provides an alternative method of decompressing a lumbosacral plexus injury. To our knowledge, this is the first report of anterior osteotomy of the sacroiliac joint for the treatment of pelvic fracture malunion and nonunion.

In past decades, multi-stage surgery was firstly reported by Matta et al. and used to correct malunion and nonunion of pelvic fracture [1, 10, 17]. Nevertheless, this method requires changes in positions intra-operatively, with a prolonged operative duration reported as 4.6 to 8 h [8, 10, 18]. Simultaneously, an increased risk of posterior wound infection was observed by Rousseau et al. [10]. In our results, a mean operative duration of 4.4 h was presented and no cases of infection were documented. These observations suggested a promising application of APSJO via the LRA for the treatment of pelvic fracture malunion and nonunion.

Posterior sacral osteotomy is frequently employed for the correction of vertical sacrum displacement [17]. However, this carries a potential risk of iatrogenic nerve and vessel injury which is the major concern for most surgeons. Luo et al. [7] reported that one of nine cases experienced nerve root rupture during sacral osteotomy. Blood vessels and the lumbosacral plexus can be distinguished and protected under the LRA, and APSJO can therefore be conducted with low incidence of iatrogenic nerve and vessel injuries. Additionally, nerve decompression can be simultaneously achieved in cases with lumbosacral plexus injury [11]. Our follow-up data suggested an enhancement of functional recovery in cases both with and without lumbosacral plexus injury according to Majeed and BMRC measurements. Moreover, rotation of the innominate bone, which results in disability, is difficult to correct through isolated peri-sacroiliac joint osteotomy posteriorly. The combined anterior approaches with the ilioinguinal and modified Stoppa approach have been used for the treatment of malunion and nonunion of pelvic fractures, as described in recent case reports [19, 20]. The osteotomy was performed on the posterior part of the ilium by the ilioinguinal approach and on the anterior pelvic rim through the modified Stoppa approach. Nevertheless, displacement and deformity cannot be conducted if either the sacroiliac joint or a sacral fracture is involved in addition to lumbosacral plexus injury. As posterior deformity is frequently presented around the sacroiliac joint in pelvic fracture malunion or nonunion, the anterior combined approaches and iliac osteotomy may not be an ideal consideration for the correction of the peri-sacroiliac joint. In contrast, the APSJO was designed and performed according to the fracture classification of each patient in our study. This provided a personalized treatment for pelvic fracture malunion or nonunion.

The sacrum and ilium of the posterior pelvic ring are composed of cancellous bone, which is not suitable for direct forced reduction. Furthermore, the prolonged duration of fracture displacement post-injury and the contracture of muscles and soft tissue often pose challenges in achieving fracture reduction using conventional tools and techniques, even after effective osteotomy and soft tissue release around the osteotomy site. Hence, Starr frame was used to assist reduction procedure [12]. The lateral hemi-pelvis was immobilized on the operating table. After the LC-II Schanz screw was implanted and connected to the frame, reduction in the coronal, sagittal, and transverse plane was performed by moving the LC-II Schanz screw. Internal fixation was then performed while ensuring proper reduction was maintained.

**Fig. 4:**
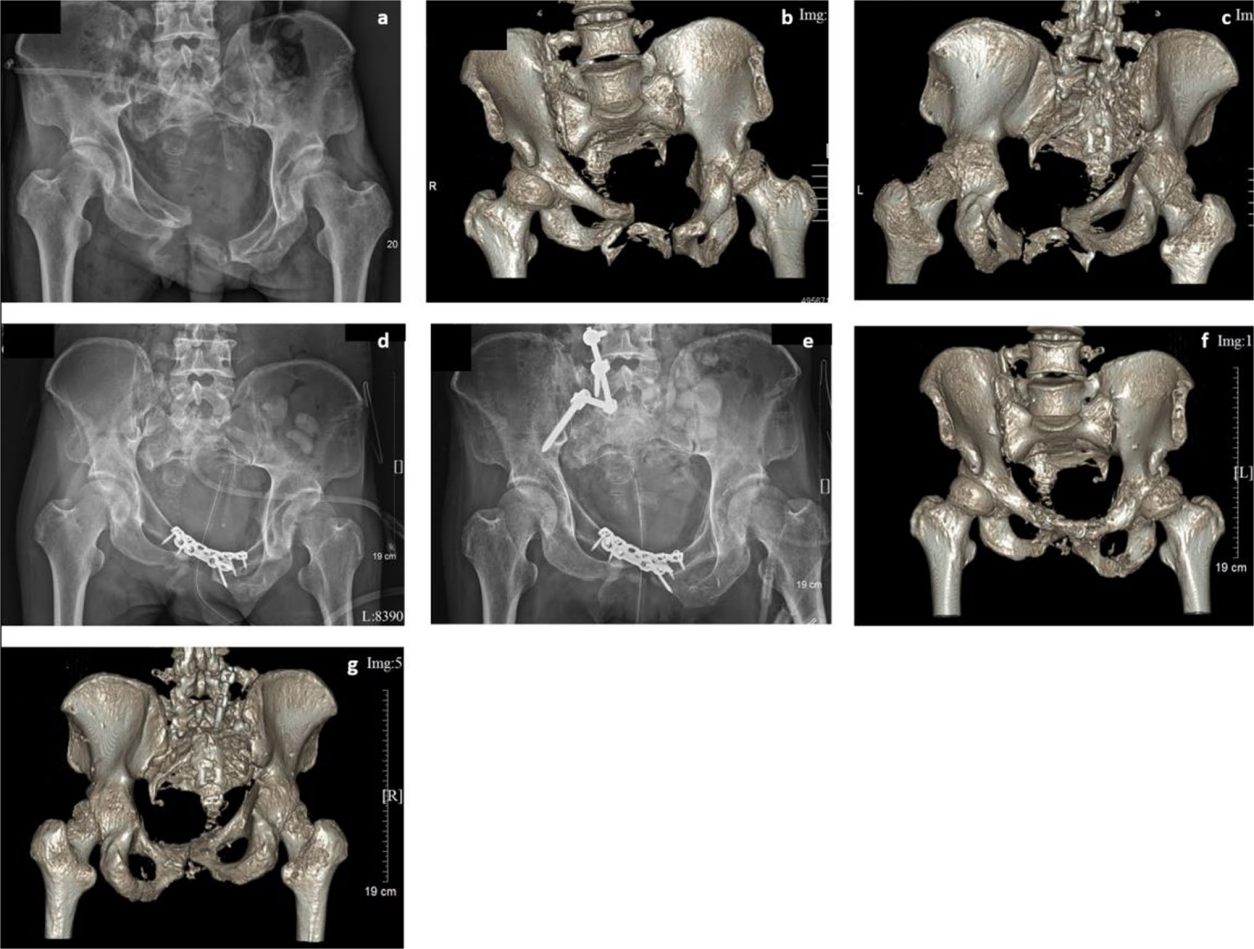
Male, 39 years old, 3 months after injury caused by a traffic accident, suffering from pain of the sacroiliac joint and pubic symphysis, with a bedsore on the sacrococcygeal region. **a**, **b**, **c** Post-operative X-ray and 3D reconstruction of CT scan showing posterosuperior sacroiliac joint dislocation and diastasis of the pubic symphysis. To avoid iatrogenic nerve injury and infection, we decided to perform a two-stage operation. **d** X-ray examination after the first stage, osteotomy of the posterior pelvic ring anteriorly through LRA without fixation; the anterior ring was fixed with reconstruction plates. **e**, **f**, **g** After 1 week of lower limb traction, the second stage was performed in the prone position; lumbopelvic distraction osteosynthesis was used in fixation

In this case series, two patients with Tile C injuries were treated by two-stage operations (Fig. 4). The first stage of surgery consisted of soft tissue release, osteotomy, and anterior ring fixation; then, reduction and fixation of the posterior pelvic rim were conducted later after two weeks of lower limb traction. The patients achieved satisfactory outcomes without any complications at the final follow-up.

In this presented case series, APSJO was used to correct the deformity of pelvic fracture malunion and nonunion through the LRA, and preliminary results demonstrated that this surgical treatment is safe and potentially effective. Although this study has limitations of a low case number and the absence of a control group, our data suggest a promising application of APSJO for the treatment of pelvic fracture malunion and nonunion. This study may provide a novel and feasible strategy for the surgical treatment of pelvic fracture malunion and nonunion.

## Data Availability

All the data used are available from the corresponding author on motivated requests.
